# Impairment of Central Chemoreception in Neonatal Rats Induced by Maternal Cigarette Smoke Exposure during Pregnancy

**DOI:** 10.1371/journal.pone.0137362

**Published:** 2015-09-02

**Authors:** Fang Lei, Xiang Yan, Fusheng Zhao, Senfeng Zhang, Qilan Zhang, Hua Zhou, Yu Zheng

**Affiliations:** Department of Physiology, West China School of Preclinical and Forensic Medicine, Sichuan University, Chengdu, Sichuan, PR China; Rajiv Gandhi Centre for Biotechnology, INDIA

## Abstract

It has been postulated that prenatal cigarette smoke exposure (CSE) increases the risk for sudden infant death syndrome. The victims of infant death syndrome suffer from respiratory abnormalities, such as central apnea, diminished chemoreflex and alteration in respiratory pattern during sleep. However, no experimental evidence on CSE model exists to confirm whether prenatal CSE gives rise to reduction of neonatal central chemoreception in *in vitro* preparations in absence of peripheral sensory feedback. The aim of the present study was to test the hypothesis that maternal CSE during pregnancy depresses central chemoreception of the neonatal rats. The pregnant rats were divided into two groups, control (n = 8) and CSE (n = 8). Experiments were performed on neonatal (0–3days) rat pups. Fictive respiratory activity was monitored by recording the rhythmic discharge from the hypoglossal rootlets of the medullary slices obtained from the neonatal rats. The burst frequency (BF) and integrated amplitude (IA) of the discharge were analyzed. Their responses to acidified artificial cerebrospinal fluid (aCSF) were tested to indicate the change of the central chemosensitivity. Under condition of perfusing with standard aCSF (pH 7.4), no significant difference was detected between the two groups in either BF or IA (*P*>0.05). Under condition of perfusing with acidified aCSF (pH 7.0), BF was increased and IA was decreased in both groups (*P*<0.01). However, their change rates in the CSE group were obviously smaller than that in the control group, 66.98 ± 10.11% vs. 143.75 ± 15.41% for BF and −22.38 ± 2.51% vs. −44.90 ± 3.92% for IA (*P*<0.01). In conclusion, these observations, in a prenatal CSE model, provide important evidence that maternal smoking during pregnancy exerts adverse effects on central chemoreception of neonates.

## Introduction

Breathing is an exceptionally reliable and fundamental physiological process that maintains life. It is generally agreed that the rhythm underlying breathing in mammals depends critically on intrinsically rhythmic pacemaker neurons in the pre-Bötzinger complex (pre-BötC) in the ventrolateral medulla [1–3]. Central chemoreceptors sense CO_2_/H^+^ levels within the central nervous system and regulate the activity of the respiratory network to stabilize brain and arterial P_CO2_/pH at physiological level. Studies have suggested that respiratory central chemoreceptors are located in the retrotrapeziod nucleus/parafacial respiratory group (RTN/pFRG) of the medulla oblongata, where the neurons express transcription factor Phox2b [4,5]. Mutations of PHOX2B cause congenital hypoventilation syndrome, a disease characterized by extremely reduced chemoreflexes [6]. RTN/pFRG neurons not only respond to increases in P_CO2_ *in vivo*, but also are activated by mild extracellular acidification *in vitro* due to their intrinsic pH-sensitivity [7].

Studies in both humans and animals indicate that exposure to tobacco smoke or nicotine during pregnancy exerts detrimental effects on the fetal development of the brainstem where the main cardiorespiratory centers are localized [8–10]. We previously reported that prenatal cigarette smoke exposure (CSE) results in apoptotic cell death in the brainstem regions that control respiratory activity [11]. More notably, maternal cigarette smoke during pregnancy increases the risk for sudden infant death syndrome (SIDS) [12–14]. Infants who succumbed to SIDS have previously shown delayed development within the central nervous system [15], blunted chemoreflex [16] and central apnea [17]. However, the relation between prenatal CSE and the diminished central chemoreception is unknown. Studies investigating the effects of prenatal CSE on respiratory control *in vivo* suggested that, at least in rodent animals, the constituents of CSE are more injurious than nicotine infusion alone as observed in other studies [18–20]. Although the effect of prenatal nicotine exposure on neonatal central chemoreception has been studied in previous works [21], nicotine infusion alone is not an equivalent model for tobacco smoking, because nicotine is just one of thousands of chemicals in cigarette smoke, some of which have neurotoxic effects. To our knowledge, no direct experimental evidence exists to confirm whether prenatal CSE exerts deleterious effects on neonatal central chemoreception in a prenatal CSE animal model.

The current study was carried out to investigate the effect of acidification on respiratory control in neonatal rats prenatally exposed to cigarette smoke instead of nicotine application, in medullary slice preparation, a model in absence of peripheral sensory feedback, to confirm the speculation that prenatal CSE directly diminishes central chemoreception.

## Materials and Methods

### Ethics statement

This study was carried out in strict accordance with the recommendations in the National Institute of Health Guide for the Care and Use of Laboratory Animals (NIH publications No.80-23) revised 2010. All experimental procedures were approved by the Sichuan University Committee Guidelines on the Use of Live Animals in Research.

### Animal grouping

Adult Sprague Dawley rats (body weight: female, 240g-260g; male, 270g-290g) were obtained from Sichuan University Experimental Animal Center. The rats, at the beginning of the experiments, were kept in a room with a 12 h light/dark cycle and had access to food and water *ad libitum*. Pregnancies were established by the overnight mating of one mature male with two nulliparous females. Pregnancy was confirmed by the presence of spermatozoa and the day was considered as gestational day 0 (gd 0). Pregnant rats were divided into two groups: control group (n = 8) and CSE group (n = 8). One or two rat pups were randomly chosen from one litter to be used in the current experiment.

### Cigarette smoke exposure

CSE were carried out from gd 7–20 as previously published [11]. In brief, pregnant rats were exposed in an inhalation chamber (80×60×50 cm) to tobacco smoke generated by lit cigarettes (Tianxiaxiu, 11 mg of tar and 1 mg of nicotine per cigarette, China Tobacco Chuanyu Industrial Co., China). The daily CSE was conducted in two sessions, one in the morning at 9:00 and one in the afternoon at 16:00. For each session, the pregnant rats were exposed to a total of 10 cigarettes over a period of 60 min: 2 lit cigarettes were allowed to burn for 10 min followed by a 2 min interval and this was repeated 5 times. Previous studies in our laboratory have determined the serum cotinine level from this protocol by ELISA detection. Using this regimen, serum cotinine concentration (92.3 ± 15.7 ng/ml) [11] achieves a level of smoking exposure that simulates active smoking during pregnancy [22,23]. The control group was treated identically to the CSE group and placed into a similar chamber, but received fresh air. None of the pregnant rats or pups became severely ill or moribund during the experiment, therefore all animals survived until the experimental endpoint. However, protocols were in place to humanely euthanize sick animals with sodium pentobarbital by intraperitoneal injection.

### Preparation of medullary slices

Transverse medullary slices were prepared from neonatal rat pups, as previously described [24]. In brief, the neonates were decapitated after anaesthetization with ether to prevent suffering. Brainstems were removed in ice-cold artificial cerebrospinal fluid (aCSF) containing the following (in mM): 125 NaCl, 3 KCl, 1.2 CaCl_2_, 1 MgSO_4_, 22NaHCO_3_, 1NaH_2_PO_4_ and 30 D-glucose. Brainstems were cut in 1800 μm coronal slices with a vibrating microslicer (MA 752, Campden Instrument, USA). Slices containing the pre-BötC and pFRG, critical sites for generation of respiratory rhythm and central chemoreception, were incubated for 30 min to 1 h at 28–29°C in a recording chamber, where they were perfused with aCSF at a rate of 4ml/min. The aCSF was equilibrated with carbogen (95% O_2_-5% CO_2_) throughout the experiment.

### Recording of fictive respiratory activity in medullary slices

Recordings of the rhythmic respiratory-like discharge from hypoglossal rootlets of the slices were performed using glass suction electrodes filled with aCSF [25]. Signals were amplified, filtered (τ = 0.001s, *F* = 1 kHz) and integrated (time constant of 50 ms) by application of a BL-420F Biological Signal Processing System (Taimeng Biotech. Co., China). The activity recorded from the hypoglossal rootlets of the medullary slices in standard aCSF (pH 7.4) had to be regular and stable for at least 20 min before a 5 min baseline was recorded. Acidified aCSF (pH 7.0) was applied to slices for another 5 min followed by a 20 min recovery period to confirm that recording parameters returned to normal. pH of aCSF was adjusted by addition of HCl or NaOH. The burst frequency (BF) and integrated amplitude (IA) of the discharges from hypoglossal rootlets were analyzed.

### Statistical analysis

The electrophysiological baseline data was compared with the data obtained during application of acidified aCSF. Differences in both the basal fictive respiration and the magnitude of responses to acidification between control and CSE groups were assessed with Student’s *t*-test for independent samples. Repeated-measures ANOVA was used to determine the significance of differences between groups induced by acidification over time. All data were presented as means ± SEM. Statistical significance was set at *P*<0.05.

## Results

### Effects of cigarette smoke exposure on basal discharge of hypoglossal rootlets in medullary slices

**Fig 1:**
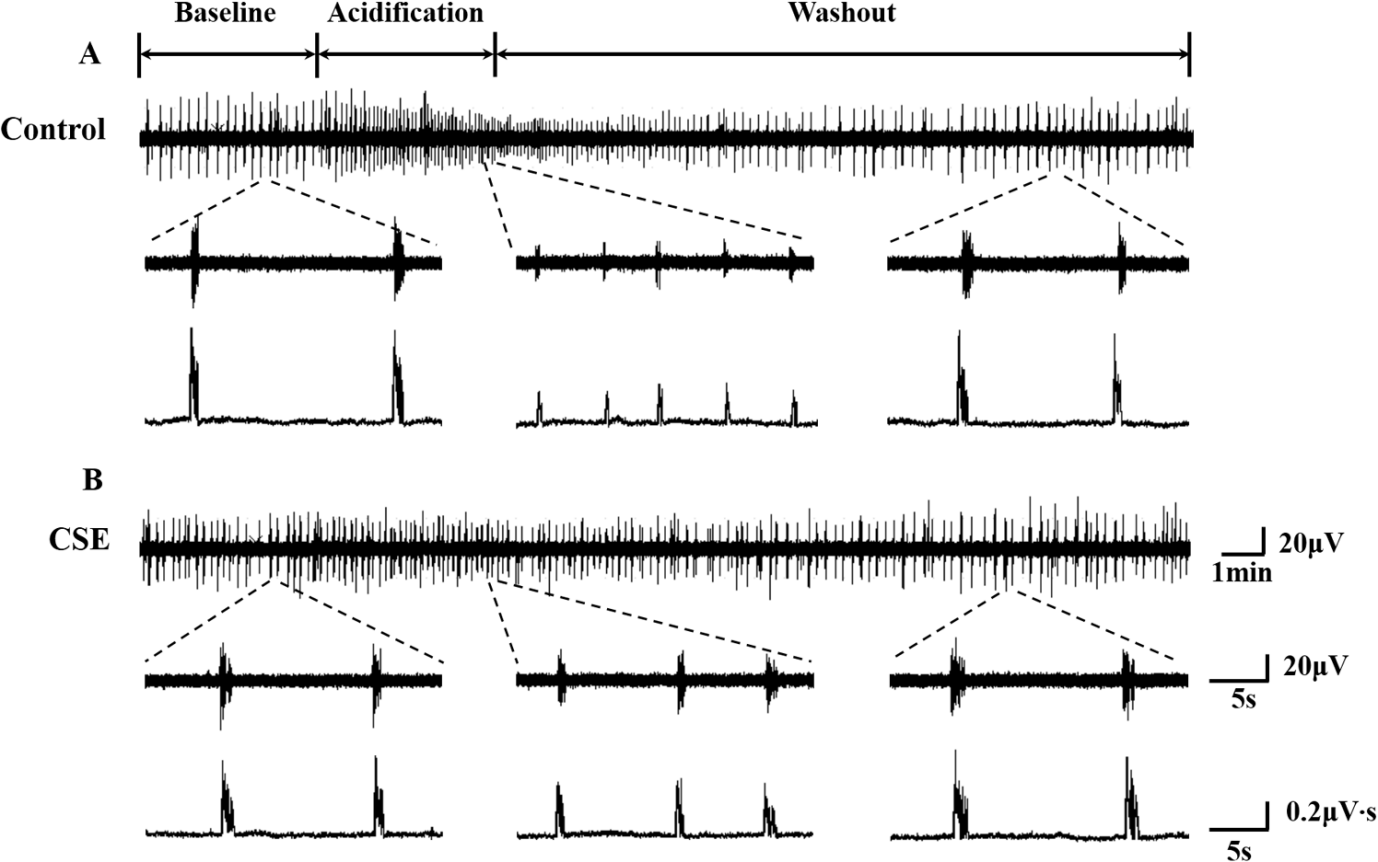
Recordings of hypoglossal rootlets discharge in medullary slices. Typical recording of the discharge from the control group (A) and the CSE group (B), respectively. In each panel, the upper line indicates the compression format of activities of the hypoglossal rootlets throughout the experiment, and the middle and lower lines indicate the raw and integrated activities of the hypoglossal rootlets before, during and after acidification, respectively.

**Fig 2:**
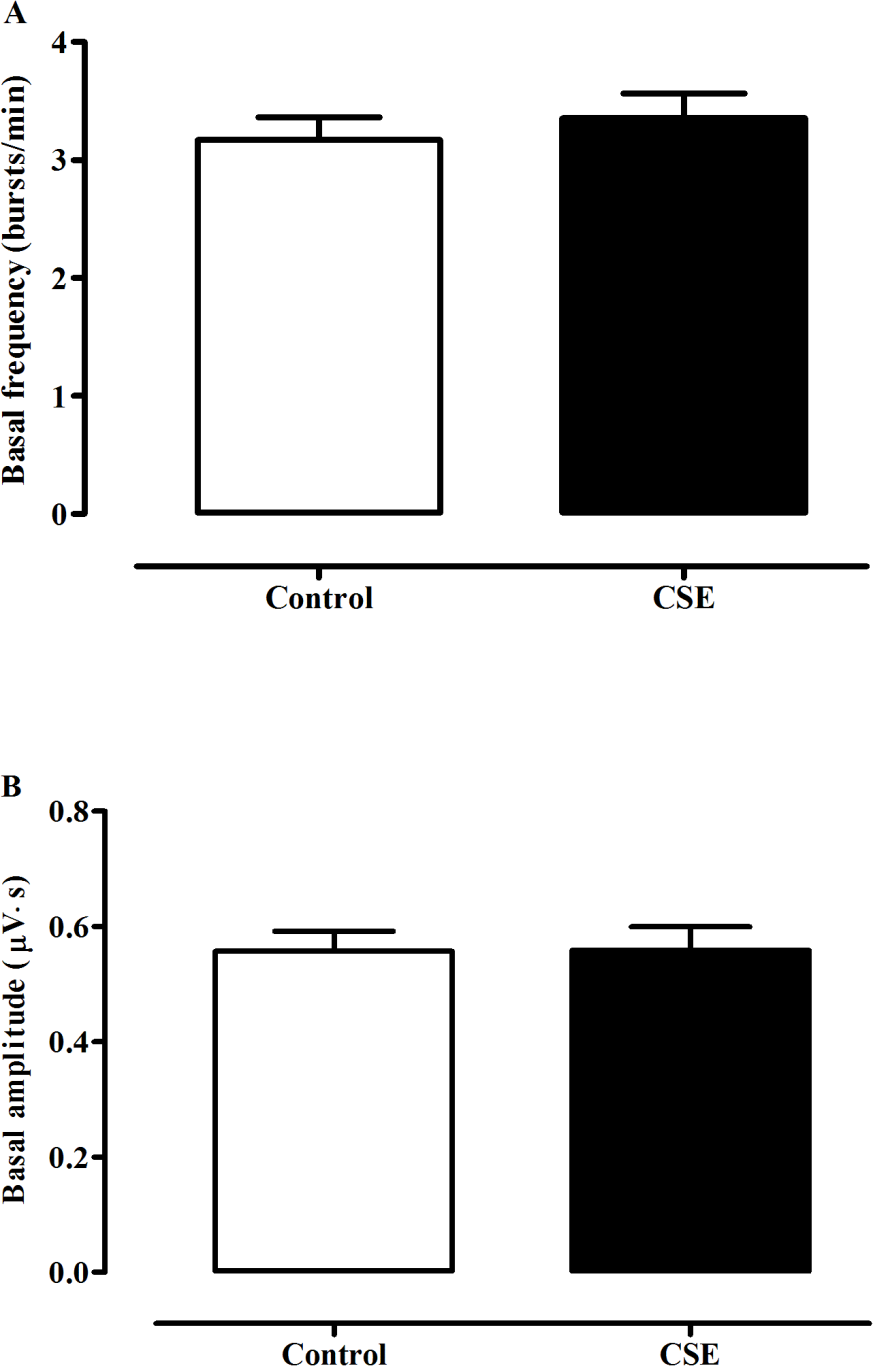
Statistical comparison of basal discharge of hypoglossal rootlets in medullary slices. Comparison of the basal burst frequency (A) and integrated amplitude (B) of the hypoglossal rootlets activities of the medullary slices in neonatal rats between the control group (n = 13) and the CSE group (n = 15).

Basal BF of fictive respiratory activity in medullary slices from CSE rat pups was 3.35 ± 0.21 bursts/min, which was not significantly different from that of the control neonates, 3.17 ± 0.19 bursts/min (*P*>0.05, Figs 1 and 2A). Meanwhile, no significant difference was detected in IA of the fictive respiration between the CSE and control groups, 0.56 ± 0.04μV·s vs. 0.56 ± 0.03μV·s (*P*>0.05, Figs 1 and 2B) when the slices were perfused with standard aCSF (pH 7.4).

### Effects of cigarette smoke exposure on responsiveness of hypoglossal rootlets discharge to acidification in medullary slices

**Fig 3:**
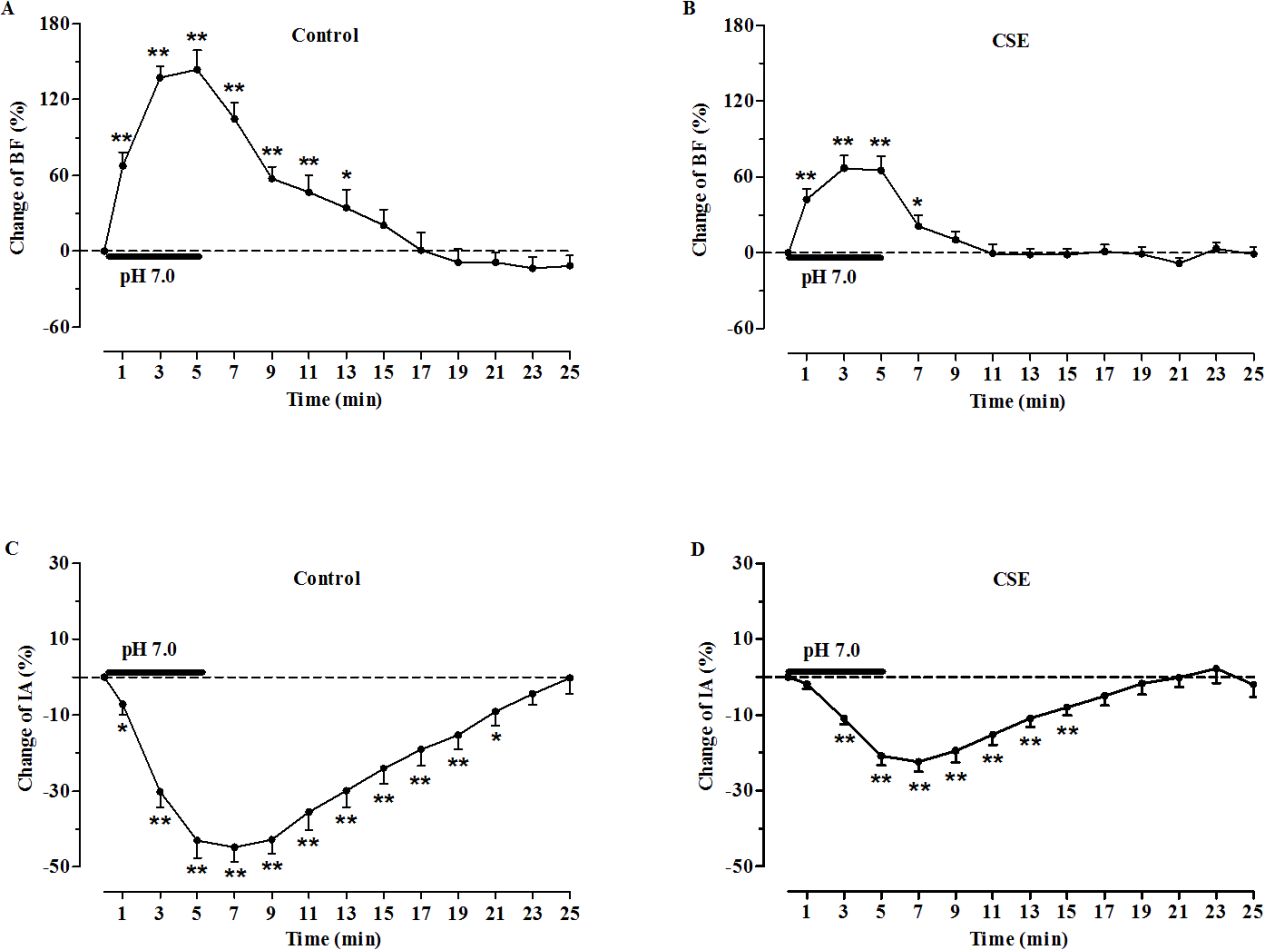
Changes in hypoglossal rootlets discharge in medullary slices induced by acidification. (A) and (B): Changes of burst frequency (BF) in the control (n = 13) and CSE groups (n = 15), respectively; (C) and (D): Changes of integrated amplitude (IA) in the control (n = 13) and CSE groups (n = 15), respectively. All data were normalized to the baseline value which was defined as the average BF and IA for 5min prior to acidic stimulation. *P< 0.05, **P< 0.01 vs. baseline.

**Fig 4:**
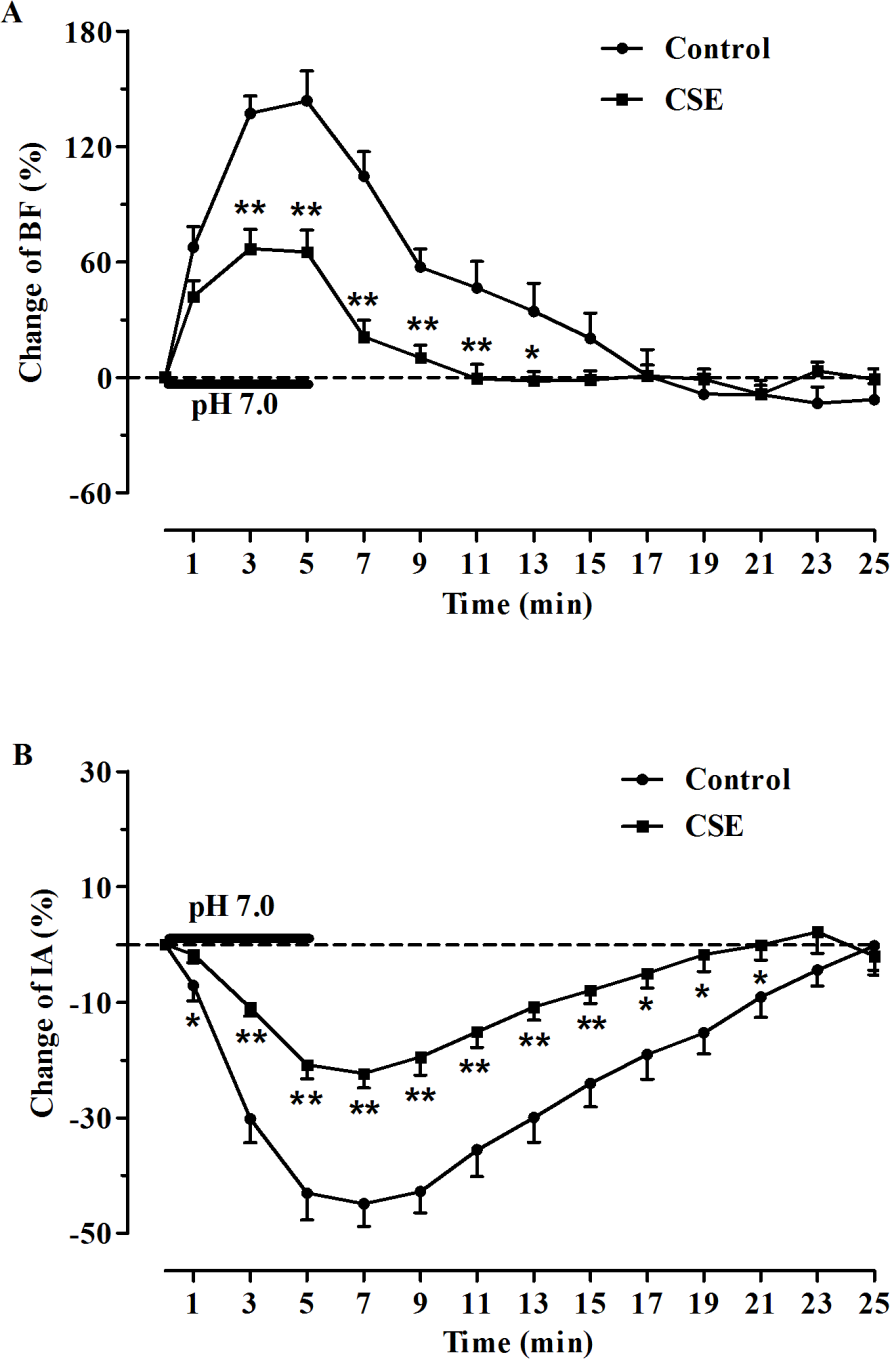
Comparison of changes in hypoglossal rootlets discharge in medullary slices induced by acidification. (A) Comparison of burst frequency (BF) of hypoglossal rootlets between the control (n = 13) and CSE (n = 15) groups; (B) Comparison of integrated amplitude (IA) of hypoglossal rootlets between the control (n = 13) and CSE (n = 15) groups. *P< 0.05, **P< 0.01 vs. CSE.

The effects of acidified aCSF (pH 7.0) perfusion on the activity of hypoglossal rootlets were observed, and the changes to BF and IA were expressed by change rates (%) compared with the basal activities. In both groups, the responses to acidification were manifested as a significant increase in BF and a decrease in IA of the fictive respiratory activity as compared with the baseline values (Fig 1). Specifically, during perfusion of the slices with acidified aCSF, the BF was increased by 143.75 ± 15.41% in the control group and 66.98 ± 10.11% in the CSE group (*P*<0.01, Fig 3A and 3B), and the IA was decreased by 44.90 ± 3.92% in the control group and 22.38 ± 2.51% in the CSE group (*P*<0.01, Fig 3C and 3D). However, the increase in BF and decrease in IA of the fictive respiration recorded from the medullary slices were reduced in the prenatal CSE rats compared with that in the control rats (*P*<0.01, Fig 4). During post-perfusion with standard aCSF (pH 7.4), the BF and IA were gradually returned to the baseline level in both control and CSE groups (*P*>0.05, Figs 1 and 3).

## Discussion

We provide direct experimental evidence that prenatal CSE has detrimental effects on the central chemoreception in neonate rats using *in vitro* preparations, in absence of peripheral sensory feedback. This study shows that prenatal CSE significantly reduces fictive respiratory responses to acidification, although it does not affect the basal fictive respiration recorded from the hypoglossal rootlets in the slices obtained from the neonates. Our study approach is different from previous animal studies which used nicotine to mimic the effects of CSE, as this model exposes animals to actual cigarette smoke. Thus our results provide some of the most direct evidence that maternal smoking during pregnancy exerts adverse impact on the central chemoreception of neonates.

The effects of maternal smoking during pregnancy have been previously investigated in human infants, whereas nicotine has been used as a replacement for prenatal CSE in a large number of animal models. Moreover, these investigations in human infants and animals have provided divergent results. Some studies in human infants observed no significant difference in respiratory drive between the infants of smokers and non-smokers [20,26,27]. Others showed that infants born to smoking mothers have a reduced drive to breath during normoxia and a diminished ventilatory response to hypoxia [28]. In contrast to these observations, another study found that ventilatory responses to hypoxia and hypercapnia were not different between the two groups, but the infants of mothers who smoked during pregnancy had a deficient arousal response to hypoxia [29]. Reasons for such contradictory results are likely the result of experimental differences in age of infants, use of sedation, extent of CSE, and composition of gaseous mixtures [18].

Animal studies also show inconsistent results. Studies in neonatal rats *in vivo* indicated that no significant effect of prenatal nicotine exposure was found on ventilation response to moderate hypoxia or hypercapnia [19,26]. However, another study showed that the ventilatory response to hypoxia was significantly attenuated during quiet sleep in nicotine-exposed neonatal lambs [30]. We previously reported that prenatal CSE did impair fictive respiratory responses to hypoxia *in vitro* in medullary slice preparations from neonatal rats [11]. Studies in neonatal mice medullary slices indicated that prenatal nicotine exposed neonates showed a reduced response to acidification only in the amplitude but not in the frequency of fictive respiration compared with the unexposed mice [21]. In this study, both basal respiratory-related frequency and amplitude recorded from the hypoglossal rootlets were not significantly different between the prenatal CSE group and the control group, whereas the responses to acidification challenge were blunted in both frequency and amplitude of fictive respiration in CSE neonatal rats compared with the control ones. These results are consistent with studies by Pendlebury et al. [18], suggesting that, in rodent animals, cigarette smoke exposure is more injurious than nicotine exposure alone to respiratory control. This is reasonable because nicotine is only one of the toxic components of cigarette smoke and many other ingredients can cross the placenta barrier to reach the fetus such as carbon monoxide. Carbon monoxide coming from cigarette smoke can easily cross the placenta barrier and inhibit the release of oxygen into fetal tissues [31]. Recurrent intrauterine hypoxic insults undoubtedly affect the development of the fetal central nervous structure, where the RTN/pFRG, the most important central chemoreceptor, is localized [4, 5].

Although the impaired central chemoreception has been postulated, the mechanisms of the disturbance induced by prenatal CSE remain unclear. Data in the present study shows that the basal fictive respiration of the medullary slices obtained from the offspring of pregnant CSE rats is not significantly different from that of control rats. This finding is in agreement with our previous work [11]. The medullary slices in our present study encompass pre-BötC which is critical for normal rhythmic respiration [3]. Loss of pre-BötC neurons leads to progressive pathological disruption of breathing and ablation of >80% of pre-BötC neurokinin-1 receptor-expressing neurons gives rise to an irregular breathing pattern [2]. We speculate that prenatal CSE does not lead to such an injury that would be sufficient to disturb normal breathing. However, the central chemoreception of the neonates was diminished after intrauterine CSE. It has been postulated that central chemoreception depends on a group of intrinsic pH-sensitive neurons and RTN/pFRG is the most critical structure, at least at birth, for central respiratory chemosensitivity [6]. These neurons express a transcription factor Phox2b which plays an important role in central chemoreception [32]. Therefore, we presume that the Phox2b positive neurons in the RTN/pFRG, retained in the thick slices used in the present study, suffered from damages caused by prenatal CSE, leading to attenuation of central chemoreception in the neonatal rats.

## Conclusion

In summary, we find that fictive respiratory responses to acidification of the neonatal rats prenatally exposed to cigarette smoke are blunted compared with those of the control rats. Together with these observations, we provide the direct experimental evidence that prenatal CSE does impair the central chemoreception of neonatal rats. Thus, the advocacy of cessation of maternal smoking during pregnancy is essential to reduce this tragedy.
